# The Efficacy of Polymer Coatings for the Protection of Electroless Copper Plated Polyester Fabric

**DOI:** 10.3390/polym12061277

**Published:** 2020-06-03

**Authors:** Alenka Ojstršek, Natalija Virant, Daryl Fox, Latha Krishnan, Andrew Cobley

**Affiliations:** 1 Institute of Engineering Materials and Design, Faculty of Mechanical Engineering, University of Maribor, Smetanova 17, 2000 Maribor, Slovenia; virant.natalija109@gmail.com; 2 The Functional Materials Research Group, Department of Engineering, Computing and the Environment, Coventry University, Priory Street, Coventry CV1 5FB, UK; ac7922@coventry.ac.uk (D.F.); ab9187@coventry.ac.uk (L.K.); aa2266@coventry.ac.uk (A.C.)

**Keywords:** PES fabric, electroless copper plating, conductive textile, polymers coatings, corrosion protection

## Abstract

The selective metallisation of textiles is becoming a very important process in the development of electronic or e-textiles. This study investigated the efficacy of polymer coatings for the protection of copper (Cu) conductive tracks electroless plated on polyester (PES) fabric against laundering and rubbing, without essentially affecting the physical-mechanical and optical properties of the base material. After the electroless deposition of a consistent layer of Cu onto PES, four polymers were applied individually by screen-printing or padding. The physical-mechanical characterisation of coated textiles revealed that polyurethane resin (PUR) and modified acrylate resin (AR) had little effect on the air permeability, tensile strength and breaking tenacity of the PES, as compared to silicone elastomer polydimethylsiloxane (PDMS) and epoxy resin (ER). On the other hand, PUR and PDMS had higher abrasion resistance and photo-stability under prolonged UV irradiation, as compared to AR and ER. In addition, freshly Cu plated samples were coated with polymers, washed up to 30 cycles and characterised by measuring their electrical resistivity, determination of colour changes and the examination of the surface morphology. Based on these results, PUR presented the most suitable protection of Cu tracks on PES, with the lowest impact on physical-mechanical properties. ER is not recommended to be used for protection of Cu tracks on fabrics, due to its rigidity, low photo-stability, washing and wear durability.

## 1. Introduction

The rapid growth in wearable technology has seen an increased demand for improved integration of electronic circuitry into textiles. The introduction of electrical conductivity onto the surface of textiles leads to the production of high-added value textiles i.e., the dubbed electronic textiles (e-textiles), with applications in electronic sensors, data storage, optoelectronic and photonics. Such e-textiles have found use in diverse commercial sectors such as sport, protective clothing, healthcare and technical purposes [1]. This has often involved electronic components and circuit boards being attached to the textile, but this inevitably leads to a change in the feel and drape of a textile and increases its mass. With its high conductivity and ductility, copper (Cu) is one of the most attractive options for e-textiles and application on the surface of flexible, substrates. Electroless Cu deposition is a well-known, additive approach to coat non-conductive (dielectric) substrates, such as printed circuit boards [2], textile [3], glass [4,5] and ceramics [6], with a conductive, metallic layer. Electroless plating is a controllable, stable process, which includes a source of metal ions, reducing agents, complexing agents, a stabilizer, a buffering agent, and a wetting agent; with temperature, concentration of the reducing agent and pH as the most influential factors [7]. A palladium catalysed, redox reaction takes place during electroless deposition, which reduces Cu ions at a regular rate to Cu metal, whilst formaldehyde is oxidised at high pH [2]. Once electroless copper deposition has occurred, it is essential to protect Cu tracks from oxidation that can lead to degradation of their electronic and mechanical properties in any aggressive environments they may encounter such as sweat or washing. In addition, the textiles themselves are subjected to processes and activities during their lifetime, such as washing, (tumble) drying, and flexing whilst also being exposed to a variety of conditions: humidity, extreme temperatures, irradiation by UV, rain, etc. Consequently, good adhesion between the metal layer and substrate, especially polyester, can be difficult to achieve [8,9]. The roughness of textiles can also pose a challenge to the uniform and continuousness coating, influencing the conductivity [10]. Thus, a stable protection of the coating is very important. Poor protection could cause the plated metal to peel off during wear and laundering, which limits the widespread application of wearable devices [11,12]. However, it is also important that whatever approach is used to protect the Cu, it does not have a negative impact on the physical-mechanical and optical properties of the base textile, whilst, from an economic and ecologic perspectives, it must remain cost effective and nontoxic.

Considering different methods for the corrosion protection of metallic materials, the use of organic coatings is the most widely applied approach [13]. The mechanisms of corrosion protection by organic coatings can be described by distinguishing two types of coatings: (i) barrier coating and (ii) coatings containing substances, which are active chemically or electrochemically. A number of research papers report diverse encapsulants or casting compounds for the protection of conductive layers on flexible textile materials, ensuring their reliability and long-term performance (conductivity), depending on the type of textiles, the metal being protected and the application procedures. Kazani et al. improved the washing durability (after 20 washing cycles) of two silver inks on fourteen woven fabrics (cotton, viscose, polyester, polyamide), by laminating a thermoplastic polyurethane layer over the conductive silver [14]. Yang et al. investigated ultraviolet (UV)-curable polyurethane acrylate paste as the protective layers and Paul et al. applied two UV-curable polyurethane pastes as interface and encapsulation layers on polyester fabric (PES)/cotton fabrics, by means of the screen-printing process, resulting in waterproof and durable screen printed silver on textiles [15,16]. In another approach, Cho et al. used polyurethane-sealing onto Cu/Ni electroless plated polyester fabric for e-textiles [7]. Govaert and Vanneste employed several techniques to protect a conductive layer consisting of carbon nanotubes i.e., direct coating, transfer coating and screen printing, for the application of water based polyacrylate binder [1]. To improve wear durability and adhesion at the interface between Cu film and PES fabric, Jiang et al. treated the Cu-coated polyester fabric with two commercial solutions of polyester-polyurethane and aqueous acrylate in different concentrations, which also showed advantages in the resistance to aging [17]. However, there is very little reported work on the post treatment of Cu-coated polyester fabric for the improvement of wear and washing durability.

This research is divided into two parts: (i) the application of four commonly used polymers (polyurethane, acrylate, epoxy and polydimethylsiloxane) on PES fabric using two application processes and the subsequent characterisation of the fabrics. The intention here was to evaluate the influence of individual coatings on the physical-mechanical properties of the fabric, as well as to assess the photo-stability of coatings against prolonged UV irradiation and its abrasion resistance against rubbing; (ii) the use of polymer coatings to protect electroless Cu plated PES fabric against oxidation (corrosion) after washing (up to 30 cycles) and any changes this may have on the electrical resistivity of the Cu, its colour and the effect on the surface morphology of the Cu deposit by microscopic techniques. The polymers selected for this research meet specific requirements including reasonable price, low environmental impact, wide industrial applicability, good resistance against water, low temperature performance and good adhesion on diverse types of textile materials. In this way, the suitability of the employment of particular polymers for protection of subsequently Cu metallised textiles would be determined with potential applications in wearable electronics.

## 2. Experimental

### 2.1. Materials

A series of trials were performed using an industrially bleached plain-weave 100% polyester fabric (PES) with a mass/unit area of 120.6 ± 0.89 g/m^2^ and a thickness of 0.29 mm, warp density of 69 threads/cm and weft density of 31 threads/cm.

Paraffin wax (Merck KGaA, Darmstadt, Germany) was applied to the fabric, to ensure that copper was only plated onto the desired area of the fabric. A commercially available electroless copper process supplied by A-Gas Electronic Materials Ltd., Rugby, UK was utilised involving the following products. For the electroless copper pre-treatment: Conditioner 3320A, Circuposit Pre Dip 3340 and Circuposit Catalyst 3344. The electroless Cu plating solution was made using Circuposit 3350 M-1 (M-1), Circuposit 3350 A-1 (A-1), Cuposit Z-1 (Z-1) and Cuposit Y (Y). The pre-treatment and electroless solution were prepared according to the supplier instructions.

Four commercially available polymers were selected for the protection of conductive tracks, namely, Sylgard 184 silicone elastomer kit (polydimethylsiloxane—PDMS) that contains two chemicals, base and curing agent (mixed in ratio 10:1) supplied by Dow Corning, Midland, Michigan, US: Elpeguard SL 1305 AQ-ECO (waterborne polyurethane resin—PUR): Elpeguard SL 1307 FLZ-S (modified acrylate resin —AR) and: Elpecast Wepox VT 3000 (epoxy resin—ER) solvent-free 2-pack casting resin (component A: component B, mixed in ratio 2:1), supplied by Lackwerke Peters GmbH&Co., Kempen, Germany. The main properties of these polymers are shown in Table 1.

### 2.2. Selective Metallisation of Fabric by Electroless Copper Plating

**Figure 1:**
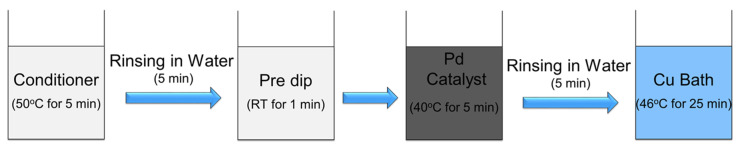
Scheme of the steps involved in an electroless copper (Cu) plating of polyester (PES) fabric.

A PES fabric sheet was sectioned into samples of size 7 × 4 cm^2^. The fabric sample was brushed with molten paraffin wax solution at a temperature of 65 °C, which left only the desired section of the textile, with an area of 4 × 4 cm^2^, exposed for Cu plating. Electroless Cu plating was conducted as outlined in the steps of Figure 1. After drying the fabric sample at RT, it was dipped into the conditioner solution (450 mL deionised (D.I.) water with 50 mL conditioner) at 50 °C for 5 min, which cleaned and functionalised the fabric’ surface. Subsequently, the sample was rinsed thoroughly with the running tap water for 5 min, and further dipped into the pre-dip solution (420 mL D.I. water with 135 g of pre-dip) for 1 min at RT. In addition, the fabric was immersed into the palladium (Pd) catalyst solution (420 mL of D.I. water with 135 g of pre-dip and 15 mL of Pd catalyst) for 5 min at 40 °C. The sample was then rinsed in water for 5 min and placed in to an electroless Cu bath, which was prepared as follows (for 500 mL): 405 mL D.I. water, 75 mL of M-1, 5.0 mL of A-1, 5.25 mL of Z-1 and 7.5 mL of Y. The bath was heated to 46 °C and the sample was immersed into the solution for 25 min. The metallised fabric was finally rinsed in D.I. water for 5 min, with the aim to remove excess plating solution, and dried in an oven (40 °C, typically overnight). The wax from the fabric was removed using ice water and then the fabric was washed in water at 60 °C, for the removal of the last traces of wax. The sample was wrapped in an aluminium foil and stored in a vacuum desiccator for characterisation.

### 2.3. Application of Polymer Protective Coatings

Two industrially applicable processes, i.e., screen-printing and padding, were used for the application of the protective coatings on textile material, due to significantly different viscosities of the four selected polymers.

PDMS and ER were applied onto fabrics using a screen-printing procedure, by means of a semiautomatic printing table Mini-MD (Johannes Zimmer, Klagenfurt, Austria). In order to achieve an even coverage of the polymer over the entire sample surface, the screen-printing frame with a PES 125 mesh stencil was placed on uncoated or metallised samples. The stainless-steel rod-roll squeegee was always rectangular on a warp direction of fabric. Pre-optimised parameters were used during printing, i.e., roll-rod diameter of 15 mm, speed of 1 m/min, max magnetic pressure and two application layers.

PUR and AR were applied onto PES during the padding procedure, according to the previously optimized method. The sample (in a warp direction) was padded twice with PUR or AR dispersion, using a two-roll laboratory pad-mangle (Werner Mathis AG, Oberhasli, Switzerland) with a horizontal position of the squeezing rollers. The padding rate was 0.8 m/min, with the squeezing roller pressure of 2 bars along the roller contact line (80% wet pick-up). The padding was repeated twice, without intermediate drying.

After the application of the polymers, coated samples were dried in an oven at a temperature of 100 °C for the time recommended by the polymer supplier (Table 1), in order to form a sufficient cross-linked network. The add-on percentage of the individual polymer on the surface of PES fabric was calculated after application according to Equation (1):

(1) $$\mathrm{Add}-\mathrm{on}=\frac{m_{c}-m_{i}}{m_{i}}\times 100\;\left(\%\right)$$

where *m_c_* is a mass of coated PES sample; and *m_i_* is a mass of PES before coating.

### 2.4. Washing

In order to evaluate the washing durability of the polymers applied onto PES, and consecutively, their protection ability against corrosion of Cu tracks, Cu-metallised and protected samples were washed up to 30 times in a Labomat (Werner Mathis AG, Oberhasli, Switzerland), according to standard ISO 105-C06, at a temperature of 40 °C for 30 min, using a solution of 1 g/L of standard reference detergent without optical brighteners, and a liquor-to-fabric weight ratio of 50:1. After each washing cycle, the samples were rinsed three times under tap water, where each rinsing cycle lasted for 1 min, and then dried at room temperature. Electrical resistivity and CIE colour measurement of the samples were carried-out before and after a selected set of washing cycles; electrical resistivity was measured after 5, 10, 20 and 30 cycles, and colour after 5, 15 and 30 cycles. In addition, the changes in surface morphologies of samples before and after the washing tests were inspected by optical microscopy (OM) and scanning electron microscopy (SEM).

### 2.5. Analytical Methods

#### 2.5.1. Characterization of Polymers’ Coated Textiles

The physical-mechanical properties, air-permeability, abrasion resistance and photo-stability of polymer coated PES fabric were determined. This part of the study investigated the effect of the four polymers on the physical-mechanical and optical properties of a base textile material. All samples were conditioned before testing for 24 h in a standard atmosphere in climatic chamber, according to ISO/R 139 at temperature of 20 ± 2 °C and relative humidity of 65 ± 5 °C.

The thickness of un-coated and coated samples was determined according to standard ISO 5084, using universal thickness gauge meter (Luis Schoppen, Leipzig, Austria). Each sample was measured five times and the average value was calculated.

The air permeability of the fabric, before and after polymer coating, was carried out on five different places on each sample, according to standard EN ISO 9237, using a Karl Schröder KG device (Weinhem, Germany).

Selected mechanical properties, such as elongation at break, tensile strength and breaking tenacity of uncoated and polymers’ coated samples were determined, according to Standard ISO 13934-1, using a Textechno statigraph M test machine (Textechno H. Stein GmbH & Co. KG, Moenchengladbach, Germany). A total of five measurements were taken separately in both weft and warp directions for each sample (25 × 5 cm^2^), in order to obtain statistically significant results.

The abrasion resistance of polymers’ coatings on fabric was accomplished by the Martindale method, according to standard EN ISO 12947-3, using up to 20,000 rubbing cycles. The mass loss of the samples was determined after each set of rubbing cycles (5,000, 10,000 and 20,000), according to ISO 3801 standard, using a Zweigle KG device (Zweigle Textilprüfmaschinen GmbH & Co KG, Reutlingen, Germany).

For the evaluation of the photo-stability of the protective polymers, selected samples were irradiated with an artificial UV source for a prolonged time (up to 30 days). Sample sizes of 8 × 5 cm were placed in a Luzchem photo-reactor (Luzchem Research Inc., Ottawa, Ontario, Canada), equipped with six overhead UV lamps, of which three lamps providing UV-A light within the range of 316–400 nm, with the main peak at 350 nm, and three lamps emitting mainly UV-B light within the range of 281–315 nm, with the main peak at 313 nm. The UV luminance in the reactor was 18,600 Lux, measured by a Sperry Instruments Digital Light Meter, SLM-110 (ECM Industries, LLC, New Berlin, USA). The time-dependent stabilities of the applied polymers towards UV-provoked changes in fabrics’ whiteness/yellowness were calculated after 30 days of irradiation (Equations (2) and (3)) from the reflectance measurements obtained by two-rays spectrophotometer Spectraflash SF 600 PLUS (Datacolor, Lawrenceville, New Jersey, USA), as described in Section 2.5.2.

*W* = *Y* + 800 · (*x_n_* − *x*) + 1700 · (*y_n_* − *y*) (2)

where *W* is the whiteness according to CIE; *Y* is the tristimulus value; *x* and *y* are the chromaticity coordinates of the observed white sample; and *x_n_* and *y_n_* are chromaticity coordinates of the completely opaque standardized white object.

(3) $$YI=\frac{100\;\cdot \;\left(C_{X}\;\cdot \;X-C_{Z}\;\cdot \;Z\right)}{Y}$$

where *YI* is the yellowness indices according to ASTM E313 method at D65/10°; *C*_X_ and *C*_Z_ are coefficients, depend on illuminant and observer; *Y*, *X* and *Z* are tristimulus values in CIE colour space.

#### 2.5.2. Analysis of Conductive Textiles

Electrical resistivity, reflectance and colour of Cu metallised/polymer coated PES fabrics samples were determined after 30 washing cycles and the surface morphology was inspected, in order to study the suitability of selected polymers for the corrosion protection of Cu tracks on textiles.

**Figure 2:**
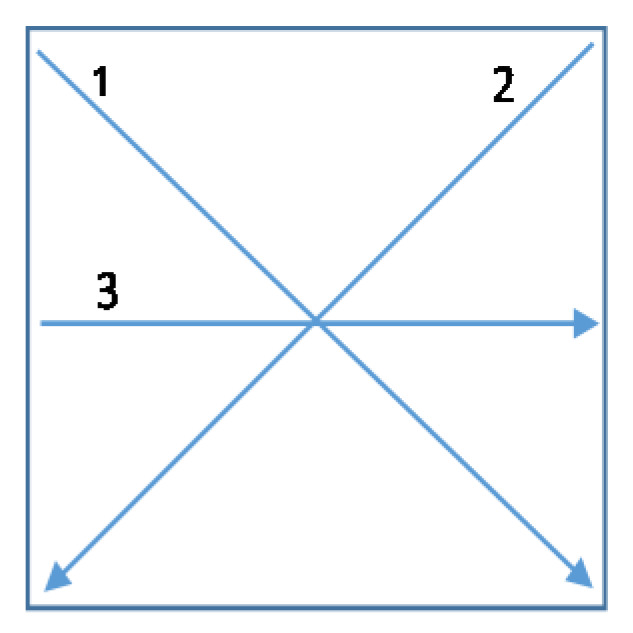
Electrical resistivity measuring lines/distances; 1 (5.7 cm), 2 (5.7 cm) and 3 (4 cm).

The electrical resistance of Cu-metallised and Cu-metallised/protected samples was measured with the use of a set of standardized measuring electrodes at three measuring points/distances on each test sample (Figure 2), using a 34410A 6 ½ Digital Multimeter (Agilent Technologies, Santa Clara, USA).

Reflectance measurements of metallised and metallised/polymer coated samples were achieved within a spectral range of 400–700 nm wavelengths, by a two-rays spectrophotometer Spectraflash SF 600 PLUS (Datacolor, Lawrenceville, New Jersey, USA), equipped with an Ulbricht sphere and measuring geometry of d/8° under a standard illuminant D65 (LAV/Spec. Incl.), from which the CIE L*a*b* colour values were calculated, using Datacolor QC 600, V3.3 software. In addition, the CIE total colour differences (dE*) of Cu metallized/polymer coated samples before and after washing cycles were calculated from the coordinate differences in all three directions of the colour space by following Equation (4) [18]:

(4) $$dE*=\sqrt{{\left(dL*\right)}^{2}\;+\;{\left(da*\right)}^{2}\;+\;{\left(db*\right)}^{2}}$$

where dE* is the total colour difference; dL* is the difference in brightness; da* is the difference at the red/green axis; and db* is the difference at the yellow/blue axis.

The surface appearance of Cu-metallised and Cu-metallised/coated fabrics before and after 30 washing cycles, as well as the cross-sectional appearance of Cu-metallised/coated fabrics after 30 washings, were observed by Axiotech 25 HD (+pol) Zeiss optical microscope (Carl Zeiss NTS GmbH, Oberkochen, Germany), equipped with an Axiocam MRc (D) high-resolution camera. All measurements were accomplished in light transmission mode, with a halogen light as the light source, using 10× magnification.

In addition, surface morphologies of Cu-metallised and Cu-metallised/coated fabrics after 30 washing cycles were inspected by attaching approximately 1 cm^2^ of fabric sample onto an adhesive carbon tape fixed to a brass holder and observed using a Zeiss Gemini Supra 35 VP Scanning Electron Microscope (Carl Zeiss NTS GmbH, Oberkochen, Germany).

## 3. Results and Discussion

### 3.1. Characterization of Polymers’ Coatings

**Figure 3:**
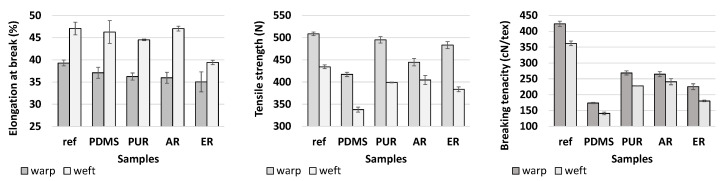
Mechanical features of uncoated and coated samples in warp and weft directions.

**Figure 4:**
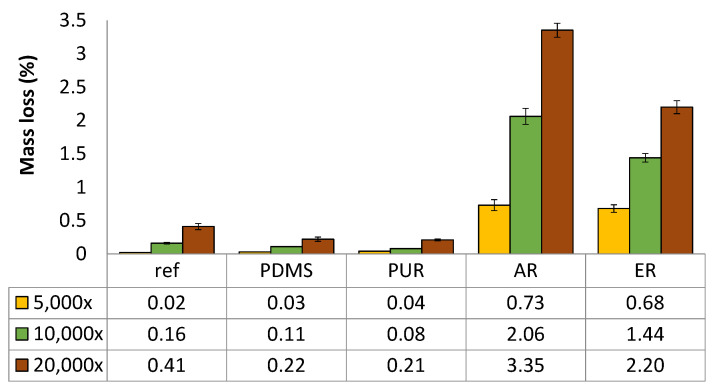
Mass loss of polymer coated PES samples, measured at 5,000, 10,000 and 20,000 rubbing cycles.

The aim of the first part of the study was to determine if four polymers, selected for their ability to protect copper from corrosion, would influence the physical-mechanical properties of the base textiles. As stated in the methodology, the PES fabric was firstly coated with an individual polymer using pre-optimized application procedures, and characterised. The obtained results are shown in Table 2 and Table 3. In addition, the durability of the polymer-coated PES against rubbing was studied by determination of the mass loss (Figure 3), and the photo-stability under prolonged UV-rays exposure by reflectance measurement, from which the yellowness and whiteness indices were calculated (Figure 4).

#### 3.1.1. Add-on, Thickness and Air Permeability

Table 2 shows the percentage of applied coatings on the fabrics, and consecutively, thickness and air permeability, depending on the type of polymer and process employed.

From Table 2, it is evident that coated samples showed weight increments between 25.38% up to 42.18%, depending on the applied polymer. AR and PUR polymers with the lower viscosities penetrate more easily into a textile structure than ER and PDMS with higher viscosities, which agrees with the results obtained by other researchers [14,19]. The air permeability of un-treated PES fabric was high, 228.4 ± 9.5 L/m^2^ s, as airflow can simply penetrate through the pores of the fabrics. The application of polymers blocked the pores between the fibres and yarns, and thus, the fabrics’ air permeabilities was reduced. This could also affect the thermal and moisture behaviour of the fabrics, if applied on larger surfaces, as suggested by Moiz et al. [20]. In our study, the highest add-on (42.18%), and consecutively, the lowest air permeability (under the limit of detection) were determined for the ER screen-printed sample. This was due to the fact that ER did not penetrate to the inner parts of the fabric, but remained on the surface, forming a thick, rigid layer, as could also be observed from the OM and SEM images. PUR and AR reduced the air permeability to a negligible extent, and therefore, they are more appropriate for the application on textiles, from the comfort viewpoint, in comparison with PDMS and ER.

#### 3.1.2. Mechanical Features

Mechanical properties are one of the important evaluations criteria of the performance of polymer coatings. It is expected that coatings tightly adhere to the fibre surface, ensuring their durability. At the same time, they should possess strength and flexibility for providing protection and extensibility. In this research, elongation at break, tensile strength and breaking tenacity of coated samples were compared with reference to uncoated PES, in both warp and weft directions. This enabled an evaluation of any possible negative effects of the employed polymers on the fabrics’ mechanical properties. The obtained results were disclosed in Figure 3.

The consideration of Figure 3 indicates that, after application of polymers, the elongation at break and tensile strength were decreased moderately, compared to the reference sample, i.e., elongation at break by 3–11% (warp) and 0.04–16% (weft), and tensile strength by 3–18% (warp) and 7–22% (weft). This could be explained by the fact that the coating penetrates the fabric sample and inhibits the mobility of the yarns in the fabric structure, causing the fabric to become more rigid and inflexible [21,22]. The fabric rigidity depends also on the characteristics of the polymers used, the application conditions, and undesired linkages between the coated fibres, so-called bridges. In addition, the breaking tenacity drastically decreased, especially using PDMS as a coating polymer (for 59%). Due to the restriction of warp and weft yarn movement in fabric structure, the fabric breaks as a whole (the yarns break at one time), as explained in detail by Bulut and Suülar [22].

#### 3.1.3. Abrasion Resistance

The durability of textiles against rubbing is a significant parameter in everyday life (abrasion between textile and skin, textile and textile, textile and surrounding material), and it is therefore important that it should be determined. Moreover, the mechanical damage of polymer coatings could lower the protection of Cu on the surface of textile material, leading to oxidation of the Cu deposit. Thus, a standard abrasion resistance Martindale method was used, where PES samples coated with the selected four polymers were exposed to 5,000, 10,000 and 20,000 rubbing cycles. The mass loss after each set of rubbing cycles was determined (Figure 4).

From Figure 4, it is observed that uncoated PES (ref) had very good abrasion resistance, as expected [23], although the mass loss increased, with increased rubbing cycles (up to 0.41% after 20,000 rubbings). By the repeated rubbing on the textile’s surface, the fibres are bent and shifted in the rubbing direction. As the number of rubbing cycles rises, deflection of the fibres increases, as well as the tension, until the point when the filaments finally break [24]. Modification of the fabric by coating with a polymer could change the slip resistance or the friction resistance, and thus, change the fabric’s abrasion resistance, depending on the type of coating compound. In this study, the highest mass loss was observed with the AR-coated sample, from 0.73 (10,000 cycles) up to 3.35% (20,000 cycles). The lowest mass loss was recorded at the PUR-coated sample (0.04% up to 0.21%), indicating a good protection of the fabric surface against rubbing. As explained in [25], PUR offers an outstanding abrasive wear resistance when compared with rubbers, plastics, or even metals, and is therefore widely used in the systems where a high abrasion resistance is required. In the case of the PDMS screen-printed sample a mass loss of up to 0.22% was observed. Although it was noted that some of the PDMS coating had peeled off during the rubbing test, it remained glued together with the textile surface in the form of beads and therefore the mass was not significantly changed. According to the literature [24], seat covers for furniture in private use should withstand 15,000 rubbing cycles without any damage, while those used in workplaces should last for at least 35,000 cycles. Thus, all the polymers from our study could be used as protective coatings, on furniture at least.

#### 3.1.4. UV Photo-Stability

**Figure 5:**
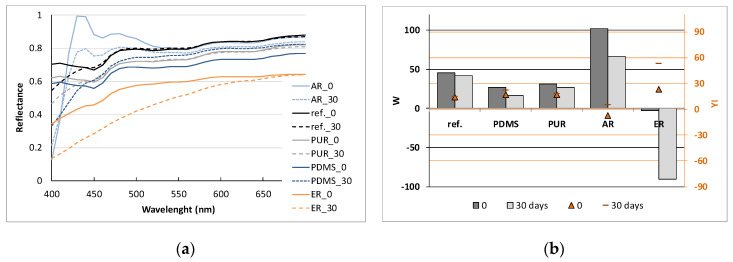
Samples before 0 and after 30 days of UV light exposure: reflectance curves (**a**) and *W* and *YI* values (**b**).

Although polymers are believed to be “everlasting materials”, they experience some type of degradation during their service life, resulting in an appreciable modification in their properties. Thus, uncoated and PDMS-, PUR-, AR- and ER-coated PES samples were irradiated in an UV chamber with a combination of artificial UVA and UVB lights up to 30 days, in order to study the photo-stability of coatings when applied on the textile material under prolonged UV light exposure. The optical changes of the samples were evaluated before and after irradiance by reflectance measurement, from which CIE whiteness (*W*) and yellowness (*YI*) indices were calculated (Equations (2) and (3)) and presented in Figure 5.

From reflectance curves (Figure 5a) and *W* and *YI* values (Figure 5b), it could be observed that after ER and AR application, the optical properties of PES drastically changed. ER is yellowish in colour and, despite its transparency and thin application layer, this caused changes in its surface reflectance, as expected.

After several days of UV exposure, ER coated sample became brownish (data not shown), escalating the *YI* from 23.15 up to 52.88 in 30 days. This was in contrast to the AR coated sample, which became whiter compared to the reference, due to the content of the fluorescent pigment, which absorbs light in the UV region (wavelengths below 400 nm) and reflects it from the surface in the Vis region above 400 nm (Figure 5a). After 30 days of UV exposure, the whiteness of the AR sample showed a very significant decrease from 102.1 down to 66.5. The application of PDMS and PUR on the PES fabric also changed the reflectance curves (Figure 5a), where the samples became darker in comparison to the reference one. However, after UV irradiation, the reflectance and *W* and *YI* values remained almost the same, which is in agreement with the results obtained by other researchers [26]. Some small changes could be observed between 400 and 440 nm, but this was also seen for the reference sample. From the obtained results, it could be concluded that both PDMS and PUR are photo-stable polymers, which are not prone to ageing, and as such, they would be appropriate for the protection of conductive tracks that would be exposed to the sun or artificial lights.

### 3.2. Characterisation of Cu Metallised/Polymer Coated PES

**Figure 6:**
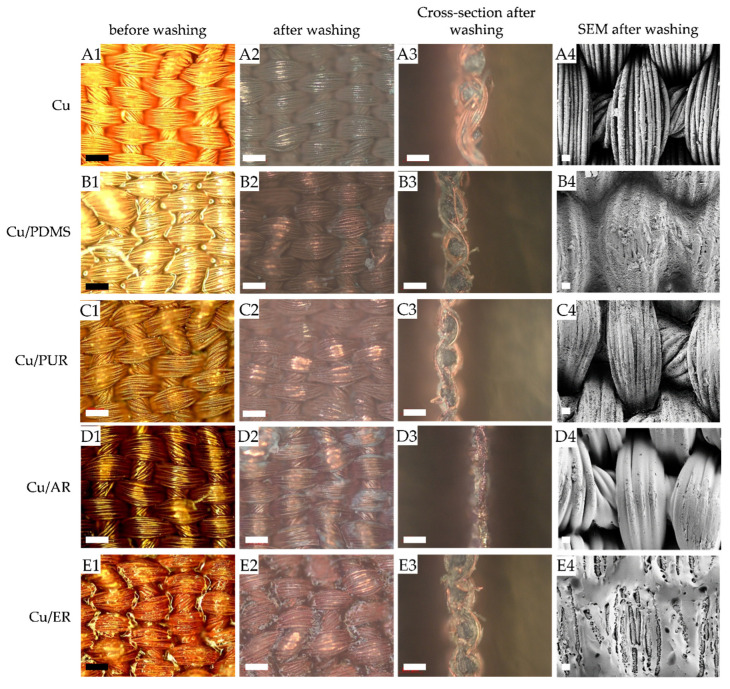
OM of Cu metallised/protected samples: before washing (**A1**–**E1**); after washings (**A2**–**E2**); cross-section after washings (**A3**–**E3**); and SEM after washings (**A4**–**E4**); Cu (**A1**–**A4**); Cu/PDMS (**B1**–**B4**); Cu/PUR (**C1**–**C4**); Cu/AR (**D1**–**D4**); Cu/ER (**E1**–**E4**). Scale bar: 200 µm for OM and 20 µm for SEM.

To evaluate the suitability of the selected polymers to protect electroless plated Cu on PES textiles against corrosion, Cu metallised/polymer coated samples were washed for up to 30 cycles and analysed using several complementary approaches. These were: (i) determination of electrical resistivity before and after 5, 10, 20, and 30 washing cycles (Table 3), (ii) investigation of optical properties by measuring the fabrics’ reflectance before and after 5, 15, and 30 washing cycles, followed by the calculation of CIE total colour differences (Table 4), and (iii) evaluation of surface morphologies before and after 30 cycles by means of OM and SEM (Figure 6). Metallised/polymer coated textiles will be further used to produce assistive technology for older adults (ongoing research).

#### 3.2.1. Electrical Resistivity

Electrical resistance of material indicates how strongly the material opposes the flow of electric current through it. In the case of conductive textiles, the resistance is affected by the uniformity of electroless copper deposit, the arrangement of fibres and yarns in the textiles, the geometrical dimensions of the sample and on its structure [27]. The electrical resistivity of samples was measured between two points at three measuring lines/distances (1, 2, 3) on each sample, as presented on Figure 2, which allowed the assessment of the conductive properties of the whole textile sample surface. Each sample was measured after electroless copper deposition and then again after protection (Cu metallised/polymer coated) and after the 5th, 10th, 20th and 30th washings (Table 3).

It should be noted from Table 4 that the electrical resistances of the Cu metallised samples were in the range from 0.1 up to 1.5 Ω, suggesting good conductivity of samples. It is also apparent that the electrical resistivity varied for the same sample, depending on the measuring line/distance, since, compared with other engineering materials, textiles are not homogeneous and anisotropic products. Specific surface and physical-mechanical properties of the textile material, such as elasticity, bending and stretching, influence the uniform coating of the electroless Cu deposit across the whole surface.

As explained by Tokarska et al. [27], the sample resistance is associated with contact resistance between neighbouring yarns and numerous contact points where yarns in the sample cross (different number of warp and weft yarns per length unit). Depending on where the electrodes are located, current flows in a different way (the path of easiest flow) in the fibrous structure. Table 3 shows that after application of the polymers, electrical resistivity increased (conductivity decreased), depending on the initial electrical resistivity, measuring line/distance, and employed polymer. The reference sample, which was not protected with polymer, recorded the highest electrical resistivity after 10 washing cycles (up to 1 MΩ), and thus, it was no longer conductive. The same trend was observed at the ER protected sample after 10 washing cycles, where the electrical resistivity was measured at 65.3 kΩ, revealing the least effective protection against water as compared with other polymer’ protected samples. During washing, the ER film formation on the surface cracked, as could be further seen from the SEM micrograph (Figure 6), and the water easily penetrates between the pores, causing a loss of conductivity. The three remaining samples were still conductive after 30 washing cycles, in the following order from the highest to the lowest: AR, PDMS and PUR, providing successful protection of the Cu metallised surfaces against water, detergent and mechanical forces during washing.

#### 3.2.2. Colour Evaluation of Samples

Samples were calorimetrically evaluated by measuring the reflectance, from which the CIE L*a*b* values were calculated. This allows for an analysis of how the optical properties are affected by the coatings, as well as the effect of laundering cycles on possible colour changes (eventual corrosion). Furthermore, the colour differences in lightness (dL*), on a*-b* axis (da* and db*) and total colour differences (dE*) were determined between Cu metallised and Cu-metallised/protected textiles plus between the non-washed and washed samples, after 5, 15 and 30 washing cycles (Table 4).

It is evident, from Table 4, that each of the five electroless Cu plated samples had a slightly different colour (a* and b* axes), as well as lightness (L* axis). After the application of the protective coatings, the lightness drastically decreased, irrespectively of the polymer used, implying darker hues, as reported in a previous study [17]. Moreover, all samples became less red and less yellow (greener and bluer) in comparison to the unprotected fabrics, as could also be perceived from the OM images (Figure 6) and determined from the reflectance values in Figure 5a. After 5 washing cycles, the most significant changes in hue (where the sample became more greenish-blue) and lightness occurred on the Cu-metallised sample that was not protected with polymer; i.e., dE* was 13.47, revealing that corrosion had already started. On the other hand, the dE* of PDMS, ER, PUR and AR protected samples increased to 2.41 after 5 cycles, up to 3.41 after 15 cycles and up to 4.41 after 30 cycles, depending on the employed polymer. These changes are not linear, and they are dependent on the uniformity of Cu deposition and polymer coatings, as well as the location of the measuring area on an individual sample.

#### 3.2.3. Surface Observation by OM and SEM

The surfaces, cross-sections, and surface morphologies of Cu-metallised and Cu-metallised/polymer protected samples, before and after washings, were inspected in detail by OM and SEM techniques (Figure 6).

Figure 6A1–A4 displays a uniform and dense Cu deposit on the surface of PES fabric obtained by electroless plating, wrapping the entire surface of an individual fibre. The polymers changed the surfaces of the fabrics in that they became smoother, although the Cu deposit is still visible, because of the transparency of the colourless polymers. It is also evident from OM and SEM images that PDMS and ER filled the pores between fibres, forming thicker films on the surfaces as compared to PUR and AR coatings, which agrees with the results in Table 2. In addition, all polymers remained on the surfaces of samples after 30 washing cycles (as shown in Figure 6A2–E2,A4–E4)), although some deterioration/cracking of the polymer film can be observed, especially at ER, due to the combined function of water, auxiliaries and mechanical forces. In general, all polymers on the coated samples become visually less bright after intensive washings as they are slightly shaded, but still yellow-reddish in comparison to the corroded green-blueish un-coated reference, as also confirmed by colour measurement (Table 4). AR presents the most homogeneous coating after 30 washing cycles (Figure 6D1‒D4), exhibiting the best protection of the Cu deposit, which agrees with the electrical resistivity results presented in Table 3.

## 4. Conclusions

In the first part of this study, four commercially available polymers were applied on PES fabric according to pre-optimised screen-printing or padding processes and comprehensively characterised, in order to evaluate their effect on the physical-mechanical properties of the base textile. Moreover, the wear and photo resistances of polymer coatings were evaluated. The results obtained by the physical-mechanical characterisation of coated PES demonstrated that PUR and AR had less influence on air permeability, tensile strength and breaking tenacity when compared to PDMS and ER. On the other hand, PUR and PDMS had the highest abrasion resistance, with mass loss of 0.21% and 0.22% at 20,000 rubbing cycles, and superior photo-stability under prolonged UV irradiation in comparison with AR and ER.

In the second part of the study, a PES fabric was successfully Cu coated using a Pd-catalysed Cu electroless plating process, as confirmed by SEM images and electrical resistivity measurement. This electroless Cu deposit was then protected using the four selected polymers. The results demonstrated that AR, PDMS and PUR imparted durable protection to Cu against 30 washing cycles, while simultaneously retaining good conductivity of the fabrics. It was found that the colour of the deposit did not change noticeably, thereby implying good corrosion protection. When considering the results of all examined parameters, PUR applied by pad-drying technique presented the most suitable protection of Cu tracks on PES, with the lowest impact on physical-mechanical properties of base textiles. On the other hand, ER, which is commonly employed in the printed circuit boards industry, is not recommended to be used for protection of Cu tracks on fabrics, due to its rigidity, low photo-stability and washing durability. The obtained results are a part of an ongoing project, and will be further used in the production of assistive technology for older-adults in 3 specific areas, namely (i) footwear: insoles that can analyse gait and warn of falls, (ii) furniture: sofa with the ability to monitor breathing and activity and (iii) clothing: t-shirt with integrated heaters that can detect body temperature and warm the user if they are cold.

## Figures and Tables

**Table 1:** Properties of applied polymers *.

Polymer	Colour	Transparency	Viscosity (m Pa/s)	Cure Time		Pot Life (h)	Dielectric Strength (kV/mm)
				At 25 °C (h)	At 100 °C (min)		
Polydimethylsiloxane (PDMS)	clear	high	3500	48	35	up to 2	19
Polyurethane resin (PUR)	colourless	high	30	4	20	up to 2	75
Modified acrylate resin (AR)	fluorescent	high	low	-	5	1	60
Epoxy resin (ER)	yellowish	high	1500	72	45	1.5	45

* Data provided by the supplier (Technical reports).

**Table 2:** Add-on, thickness and air permeability of coated PES.

Sample	Process Employed	Drying		Add-on (%)	Thickness (mm)	Air Permeability (L/m^2^ s)
		*T* (°C)	Time (min)			
reference	-	-	-	-	0.289	228.4 ± 9.5
PDMS	Screen-printing	100	25	39.26	0.323	47.8 ± 2.4
PUR	Padding	100	20	25.38	0.314	215.4 ± 13.6
AR	Padding	100	5	28.23	0.318	194.5 ± 12.2
ER	Screen-printing	100	45	42.18	0.346	˂ 5

**Table 3:** Electrical resistivity before and after washings.

Electrical Resistivity [Ω]																		
Cu Metallized			Cu Metallized/Polymer Coated				Washing Cycles											
							5			10			20			30		
**1**	**2**	**3**	**Polymer**	**1**	**2**	**3**	**1**	**2**	**3**	**1**	**2**	**3**	**1**	**2**	**3**	**1**	**2**	**3**
0.4	0.2	0.1	**-**				20.5	37.4	35.5	46.2 k	1 M	68.7 k	-	-	-	-	-	-
0.8	0.4	0.3	**PDMS**	1.2	0.5	1.8	1.5	0.6	2.0	1.8	0.7	2.5	1.8	1.3	3.4	5.8	1.5	55
0.2	0.2	0.9	**PUR**	2.5	2.3	2.9	7.5	3.5	6.9	12.7	5.8	14	17.2	12.2	16	22	26	15.5
0.2	0.2	1.2	**AR**	1.7	0.2	1.5	2.5	0.4	5.8	2.8	0.8	6.1	2.8	0.8	6.5	2.9	0.9	12.2
0.3	0.6	1.5	**ER**	3.7	7.1	7.5	7.8	5.9	2.7	11.8 k	17.4 k	65.3 k	-	-	-	-	-	-

**Table 4:** CIE values of samples and differences between Cu metallised/protected non-washed and washed samples (up to 30 cycles).

CIE L*a*b* Values							CIE Colour Differences											
Cu Metallized			Cu Metallized/Polymer Coated				Washing Cycles											
							5				15				30			
L*	a*	b*	**Polymer**	L*	a*	b*	dL*	da*	db*	dE*	dL*	da*	db*	dE*	dL*	da*	db*	dE*
42.98	14.88	13.01	-				−8.50	−7.44	−7.35	13.47	−8.26	−6.96	−6.02	12.37				
43.29	14.82	12.91	PDMS	32.67	8.11	7.48	0.53	−1.58	−0.77	1.84	−0.47	−2.90	−1.16	3.16	0.13	−4.00	−1.06	4.13
43.22	15.17	13.13	PUR	33.70	8.35	7.96	1.97	−0.42	−1.33	2.41	2.06	−0.62	−0.48	2.21	3.63	−2.01	−1.50	3.30
44.66	14.21	15.49	AR	36.25	9.82	−0.19	0.97	−0.56	0.57	1.25	−0.05	−0.88	1.68	1.90	0.52	−1.68	−2.79	4.41
44.02	14.47	12.66	ER	35.52	7.91	4.24	0.44	0.04	1.97	2.02	1.17	−1.12	2.49	2.97				
